# Antidepressant-like Effects of LPM580153, A Novel Potent Triple Reuptake Inhibitor

**DOI:** 10.1038/srep24233

**Published:** 2016-04-07

**Authors:** Fangxi Zhang, Jing Shao, Jingwei Tian, Yan Zhong, Liang Ye, Xiangjing Meng, Qiaofeng Liu, Hongbo Wang

**Affiliations:** 1 School of Pharmacy, Key Laboratory of Molecular Pharmacology and Drug Evaluation (Yantai University), Ministry of Education, Collaborative Innovation Center of Advanced Drug Delivery System and Biotech Drugs in Universities of Shandong, Yantai University, Yantai 264005, P.R. China; 2 The third hospital of Jinan, Jinan, 250132, PR China; 3 Department of Analytical Chemistry, China Pharmaceutical University, Nanjing, 210009, PR China; 4 School of Public Health and Management, Binzhou Medical University, Yantai, Shandong, PR China.; 5 ^5^Institute of Toxicology, Binzhou Medical University, Yantai, Shandong, PR China

## Abstract

The purpose of this study was to characterize a novel compound, 4-[2-(dimethylamino)-1-(1-hydroxycyclohexyl) ethyl] phenyl 3-nitrophenyl ether, designated LPM580153. We used several well-validated animal models of depression to assess the antidepressant-like activity of LPM580153, followed by a neurotransmitter uptake assay and a corticosterone-induced cell injury model to explore its mechanism of action. In mice, LPM580153 reduced immobility time in the tail suspension test, and in rats subjected to chronic unpredictable mild stress it reversed reductions in body weight gain and ameliorated anhedonia. The neurotransmitter uptake assay results demonstrated that LPM580153 inhibited the uptake of serotonin, norepinephrine and dopamine. Furthermore, LPM580153 protected the SH-SY5Y cells against the cytotoxic activity of corticosterone, an action that might be related to the role of LPM580153 in increasing the protein levels of BDNF, p-ERK1/2, p-AKT, p-CREB and p-mTOR. Together, these findings indicate that LPM580153 is a novel triple reuptake inhibitor with robust antidepressant-like effects.

Major depressive disorder (MDD) is a serious psychiatric disorder observed worldwide, palcing a tremendous economic burden with a total cost of $ 77.5–98.9 billion per year in the United States alone, and it is estimated that MDD will constitute the second highest global burden of disease by 2020^1^,^2^. Several classes of antidepressants, such as selective serotonin reuptake inhibitors (SSRIs), serotonin-norepinephrine reuptake inhibitors (SNRIs) and tricyclic antidepressants, have been prescribed for the treatment of MDD. However these antidepressants require at least 2–4 weeks of treatment to achieve therapeutic effects and have severe adverse effects, including sexual dysfunction, sleep disturbance and cardiac toxicity^3^,^4^. Furthermore, only 60% of patients respond to existing antidepressants, and as little as 30–50% of patients exhibit a full remission^5^,^6^. Therefore, more powerful drugs or novel drugs with fewer adverse effects are urgently needed in the treatment of depression^6^.

A number of studies have demonstrated the importance of dopamine (DA) in the pathogenesis of depression^7^. The mesocorticolimbic dopaminergic system has been linked to rewarding events and incentive-driven behaviours, and the reduced dopaminergic activity may lead to loss of interest and anhedonia, a core symptom of depression^7^,^8^. In a recent clinical study, combing bupropion (a DA reuptake inhibitor) with SNRIs (e.g., venlafaxine or paroxetine) administration achieved greater therapeutic effects than either drug alone^9^. Based on such findings, triple reuptake inhibitors (TRIs), which simultaneously inhibit the reuptake of serotonin (5-HT), norepinephrine (NE) and DA, have been developed as new antidepressants and have shown better efficacy and fewer adverse effects than other types of antidepressants^8^. Indeed, several TRIs (e.g. DOV21947, PRC050, TP1 and Yuanzhi-1) have advanced to preclinical and clinical trials^10^,^11^,^12^,^13^.

LPM580153 is a novel chemical entity that was designed and synthesized based on the structure of venlafaxine (Fig. 1), an SNRI with only a slight effect on the transport of DA, that has been shown to exert robust antidepressant activity in clinic practice^8^. In the present study, we demonstrate that LPM580153 reduces the immobility time of mice in the tail suspension test (TST), reverses the reductions in the body weight gain and ameliorates anhedonia in chronic unpredictable mild stress (CUMS) in rats. We also show that these antidepressant-like effects may be related to the ability of LPM580153 to inhibit the reuptake of 5-HT, NE and DA, as well as to the neuroprotective activity of LPM580153 via the regulation of BDNF/ ERK/AKT/CREB/mTOR pathways.

## Results

### Chemical structure of LPM580153 and the procedure of synthesis

LPM580153, 4-[2-(dimethylamino)-1-(1-hydroxycyclohexyl) ethyl] phenyl 3-nitrophenyl ether, was a racemate of venlafaxine derivative, and the procedure of synthesis was summarized as following: the *O*-demethylvenlafaxine was mixed with sodium methoxide and methanol, and the mixture was stirred at room temperature and concentrated under reduced pressure. The residue was azeotroped with toluene to afford off-white solid, which was then dissolved in N, N-Dimethylformamide, DMF with copper iodide, and 3-nitro-bromobenzene. The reaction mixture was allowed to stir till the thin-layer chromatography showed that all starting material consumed. The solvent was removed under reduced pressure, and the residue was diluted with EtOAc and filtered, in which the solid was washed with EtOAc. The combined organic layer was washed with 10% NaOH aqueous solution, and the organic layer was dried over Na_2_SO_4_, filtered, and concentrated. The yellowish oil was purified by flash chromatography (EtOAC:MeOH = 1:1) to afford LPM580153 as white solid.

### Effects of LPM580153 on the immobility time in the TST and the locomotor activity in the OFT

**Figure 2:**
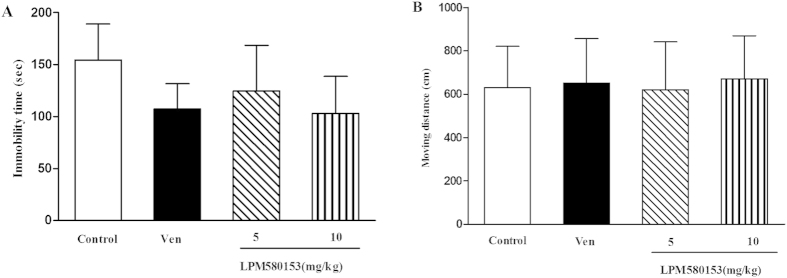
Effects of LPM580153 on the immobility time in the tail suspension test (**A**) and spontaneous locomotor activity (**B**) in mice. The animals were administered LPM580153 at doses of 5 and 10 mg/kg or Ven at 10 mg/kg by gavage 60 min prior to testing. The data were presented as the means ± SD (n = 8/group).

TST is one of the most widely-accepted animal models for predicting the efficacy of antidepressants^16^. After 60-minute oral administration, LPM580153 decreased the immobility time of mice by 19.3% and 33.2% at 5 and 10 mg/kg, respectively (F = 2.955; *p* = 0.399, *p* = 0.050), in which the venlafaxine also reduced the immobility time to some extent (*p* = 0.116). The OFT assay showed neither LPM580153 at any dosage, nor venlafaxine had a significant effect on spontaneous locomotor activity in mice (F = 0.121, *p* = 0.947) (Fig. 2B).

### Effects of LPM580153 on the body weight in CUMS rats

**Figure 3:**
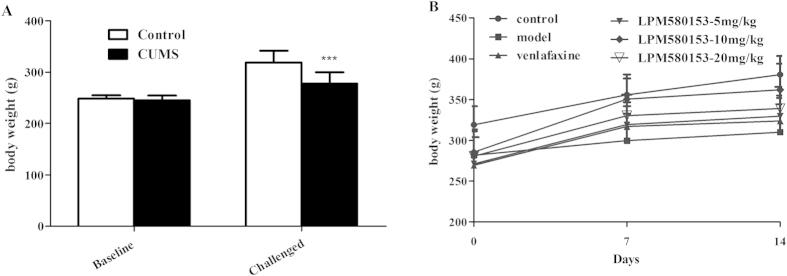
Effects of LPM580153 on the body weight in CUMS rats. The CUMS model was established, the animals were treated with LPM580153 at doses of 5, 10 and 20 mg/kg or Ven at 10 mg/kg by gavage, and then the body weight of the animals were recorded and analyzed. The data were presented as the means ± SD (n = 7/group). ***p < 0.01, compared with the control group.

CUMS can decrease the appetite of animals, reducing the body weight gain^15^. After challenged, rats exposed to 3 weeks of CUMS gained significantly less weight than those in the control group (F = 14.106; *p* < 0.001). During the two-week of chronic treatment (Fig. 3B), LPM580153 at the dosage of 10 mg/kg could significantly increase the body weight gain (F = 4.654; *p* = 0.016 at day 7; *p* = 0.019 at day 14). Venlafaxine could also reverse the reduction of body weight gain to some extent (*p* = 0.814 at day 7; *p* = 0.946 at day 14).

### Effects of LPM580153 on sucrose intake in CUMS rats

**Figure 4:**
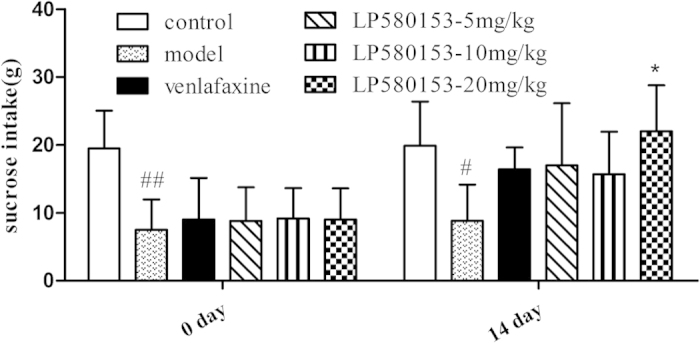
Effects of LPM580153 on the sucrose consumption in CUMS rats. The CUMS model was established, the animals were treated with LPM580153 at doses of 5, 10 and 20 mg/kg or Ven at 10 mg/kg by gavage, and then the sucrose consumption of the animals were recorded and analyzed. The data were presented as the means ± SD (n = 7/group). ^#^*p* < 0.05, compared with the control group; ^##^*p* < 0.01, compared with the control group; *p < 0.05, compared with the CUMS-model group.

The sucrose intake test is used to detect the effect of antidepressants on the anhedonia, one core symptom of depression in CUMS animals^16^. The data showed the sucrose intake was significantly lower in rats exposed to 3-week of CUMS than that in control rats (F = 12.271, p < 0.001; *p* = 0.002, Fig. 4). Treatment with LPM580153 for 2 weeks could reverse this effect, with the 20 mg/kg dose showing significant effects in comparison to CUMS-model rats (*p* = 0.038). Venlafaxine also reversed the decrease of sucrose intake (*p* = 0.394).

### Inhibition of LPM580153 on the uptake of 5-HT, NE and DA

**Figure 5:**
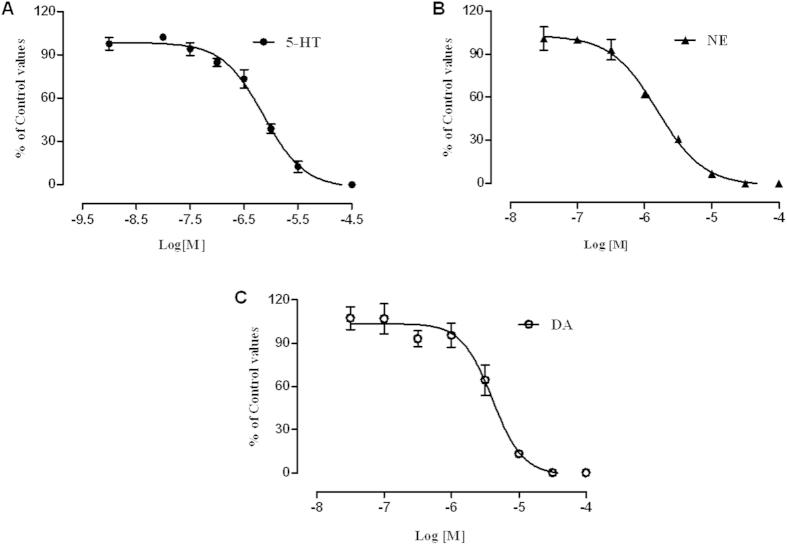
Effects of LPM580153 on the uptake of 5-HT, NE, and DA. Synaptosomes were prepared and incubated with ^[3H]^ 5-HT, ^[3H]^ NE or ^[3H]^ DA in the absence or presence of the test or reference compounds. The samples were terminated and rinsed and then detected by a scintillation counter. The data were presented as the means ± SD (n = 3, the number of independent experiments).

The effects of LPM580153 on the uptake of 5-HT, NE or DA were investigated using radiolabeled neurotransmitters. LPM580153 dose-dependently and potently blocked the uptake of ^[3H]^ 5-HT, ^[3H]^ NE or ^[3H]^ DA into the synaptosomes prepared from rat frontal cortex, hypothalamus or striatum, respectively. The IC_50_ values for 5-HT, NE and DA were 0.72 ± 0.07 μM, 1.57 ± 0.19 μM and 3.97 ± 0.71 μM, respectively (Fig. 5).

### Effects of LPM580153 on cytotoxic activity of corticosterone in SH-SY5Y cells

**Figure 6:**
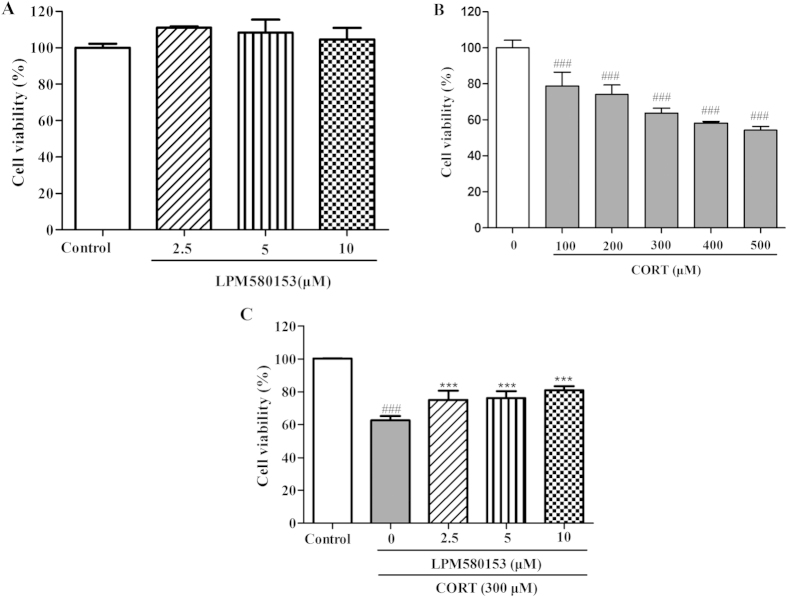
Effects of corticosterone (CORT) and LPM580153 on cell proliferation and cytotoxicity in SH-SY5Y cells. (**A**) Cell viability was determined by MTT assay after being co-cultured with various concentrations of LPM580153 for 24 h. (**B**) SH-SY5Y cells were treated with various concentrations of corticosterone for 24 h and cell survival was measured by MTT assay. (**C**) SH-SY5Y cells were exposed to corticosterone (300μM) in absence or presence of LPM580153 for 24 h and cell survival was measured by MTT assay. The data were presented as the means ± SD (n = 3, the number of independent experiments). ^###^*p* < 0.01, compared with the control group; ***p < 0.01, compared with the model group.

LPM580153 (2.5, 5 or 10 μM) treatment for 24 h exerted no cytotoxic in SH-SY5Y cells (Fig. 5A). However, after treatment for 24 h, corticosterone at the concentration of 100–500 μM could reduce cell viability in a concentration-dependent manner (F = 42.714, *p* < 0.001; Fig. 6B), among which 300 μM could reduce the cell viability to 63.6% and the concentration was used for subsequent studies. Interestingly, LPM580153 at the concentration of 2.5, 5 or 10 μM significantly attenuated corticosterone-induced damage in SH-SY5Y cells (F = 45.898; *p* = 0.010, *p* = 0.005 and *p* = 0.001, respectively; Fig. 6C).

### Effects of LPM580153 on the BDNF/TrkB-ERK/AKT-CREB/mTOR signaling pathways in corticosterone-treated SH-SY5Y cells

**Figure 7:**
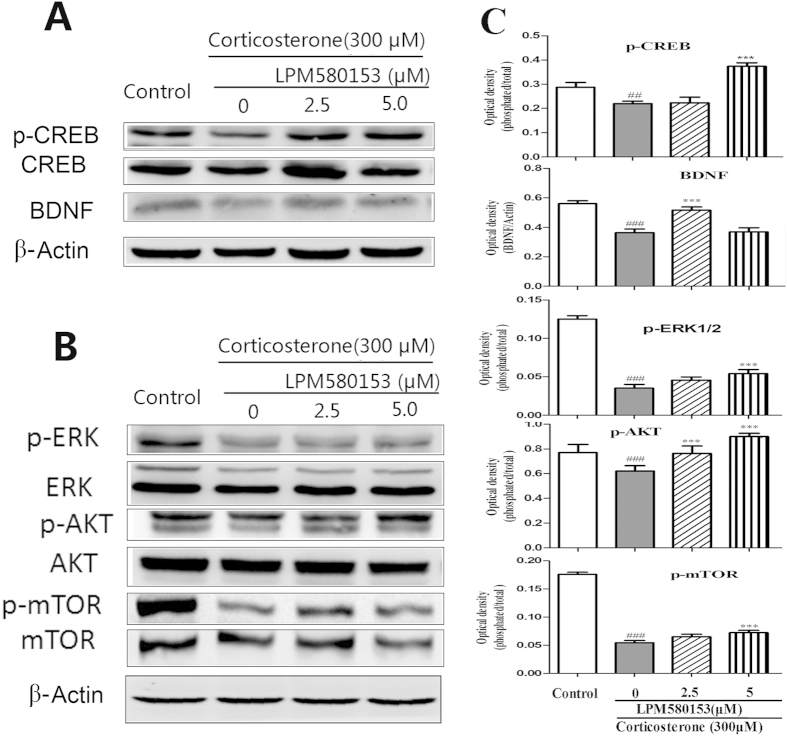
Effects of LPM580153 on inhibitory effects of corticosterone on levels of p-CREB, BDNF, p-ERK, p-AKT, and p-mTOR. SH-SY5Y cells were exposed to corticosterone (300 μM) in absence or presence of LPM580153 for 24 h, and the cells were lysate. The total protein was separated by SDS-PAGE gel under the same condition, and then the gel was cropped based on molecular weight of the target protein. The expression of the tested protein CREB/p-CREB and BDNF (**A**), as well as the ERK/p-ERK, AKT/p-AKT, mTOR/p-mTOR (**B**) were analyzed by immunoblotting using specific antibody, in which the density was semi-quantified and presented (**C**). ^###^*p* < 0.01, compared with the control group; ***p < 0.01, compared with the model group.

The effects of LPM580153 on the BDNF/TrkB-ERK/AKT-CREB/mTOR signaling pathways were explored by western blot. The data showed that corticosterone could decrease the CREB phosphorylation, which could be attenuated by co-treated with LPM580153 at 5 or 10 μM (Fig. 7A). The similar results were also noted on the expression of BDNF, as well as the phosphorylation level of ERK 1/2, AKT and mTOR (Fig. 7B).

## Discussion

MDD is a mental disorder characterized by episodes of an all-encompassing low mood and loss of interest or pleasure in normally enjoyable activities^8^. The current state of therapy for MDD, which improves depression symptoms but with a slow onset of action and poor tolerability, does not meet the medical needs of the patient population. The triple reuptake inhibitors (TRIs), which simultaneously blocked the uptake of 5-HT, NE and DA, have recently been advanced as the next generation of antidepressants. In the present study, we reported for the first time that LPM580153, a novel analogue of venlafaxine, displayed robust antidepressant-like activity in several validated animal models of depression. This antidepressant-like effect might be related to the ability of LPM580153 to inhibit the reuptake of 5-HT, NE, and DA, as well as to its neuroprotective activity mediated regulating the BDNF/ERK/AKT/CREB/mTOR pathways.

Increasing the concentration of serotonin and/or norepinephrine in the synaptic cleft, such as through the use of SSRIs and SNRIs, has been shown to be as an important and successful strategy in treating depression in clinical practices. Although such compounds display definite therapeutic effects, they also have serious drawbacks, such as limited efficacy, slow onset of therapeutic response, and adverse effects^3^,^4^. Giving the findings of clinical studies, in which combining DA reuptake inhibitors with SNRIs achieve greater therapeutic effects than either class of drug alone^9^, TRIs might be more advantageous than currently available antidepressants (SNRIs or SSRIs). Indeed, several TRIs, such as DOV21947, TP1 and Yuanzhi-1, showed much stronger antidepressant-like activity both in the preclinical studies and in the clinical trial^10^,^12^,^13^. As part of our continuing effort to discover novel antidepressant agents, we reorganized the structure of venlafaxine to generate the novel analogues, designated LPM580153. In the present study, the anti-depressant effect of this compound was firstly explored in mice using TST, a quick and well-validated assay predicting antidepressant-like activity^14^, in which single administration of LPM580153 could reduce the immobility time of animals, with effects comparable to those observed with venlafaxine. To exclude the false positive results in the TST, which are often confuse as affecting the CNS, we employed the open-field test to investigate the drug effects on locomotor activity^17^. The results showed that no significant effects of LPM580153 on spontaneous locomotor activity in mice were observed at any doses tested, indicating single LPM580153 administration indeed displayed antidepressant-like effects.

To further confirm and validate the antidepressant like activity of LPM580153, a CUMS model in rats was established. The animals were exposed to various unpredictable mild stressors to mimic the clinical symptoms of major depression in human, because the CUMS model is generally regarded as the most promising and valuable animal model of depression^18^. Indeed, the animals in the CUMS model group were observed with significantly reduced body weight gain and sucrose intake, an indicator of anhedonia, which is a core symptom of depression^16^. Chronic treatment with LPM580153 normalized body weight and increase sucrose consumption, with a potency markedly stronger than that for venlafaxine. These findings thus indicate that LPM580153 possesses much stronger antidepressant-like effects and may improve symptoms of depression.

Given the above results, the radioligand labelling methods were used to elucidate the effects of LPM580153 on the uptake of NE, 5-HT, and DA, because these neurotransmitters play important roles in the regulation of depression and have been validated as targets of several approved antidepressants. We found that LPM580153 inhibited the uptake of not only NE and 5-HT, but also of DA in the prepared synaptosomes, which indicates that LPM580153 might be a novel triple reuptake inhibitor.

The activity of the hypothalamic-pituitary-adrenal axis is disrupted in patients with depression and is characterized by elevated levels of corticosterone^19^,^20^. To mimic *in vitro* this condition of depression, we exposed SH-SY5Y cells to a high concentration of corticosterone. We found that LPM580153 markedly blocked corticosterone-induced cell injury, indicating that the antidepressant-like effects of LPM580153 might be, at least in part, related to its neuroprotective effects. To determine the mechanisms of this effect, the actions of LPM580153 on BDNF/CREB/ERK/AKT/mTOR pathways were examined in corticosterone-treated SH-SY5Y cells, and the results showed that LPM580153 increased CREB phosphorylation and increased the expression of BDNF. Furthermore, LPM580153 increased the phosphorylation of ERK1/2 and AKT, confirming that the compound activated both ERK and PI3K pathways by stimulating the BDNF pathway^21^. As a result of activating ERK and PI3K pathways, LPM580153 upregulated the level of phosphorylated mTOR. Fully understanding the underlying pathways and cross-talk, as well as the detailed mechanisms of action will require further research. However, based on the reporter that ketamine, an NMDA receptor antagonist, exerted rapid antidepressant actions by increasing phosphorylated mTOR in the rat prefrontal cortex^22^, LPM580153 might also overcome the shortcoming of antidepressants that show a slow onset of action.

In summary, our results provide the first evidence that LPM580153 is a novel TRI with robust antidepressant-like activity in several validated animal models. The antidepressant-like effect of LPM580153 may be related to both its inhibition of 5-HT, NE and DA reuptake and its direct neuroprotective activity mediated through the regulation of BDNF/ ERK/AKT/CREB/mTOR pathways. Collectively, our findings suggest that LPM580153 warrants further investigation as a potential antidepressant.

## Methods

### Chemicals and reagents

LPM580153 was synthesized by Laboratory of Medicinal Chemistry (Yantai University, Shandong, China) and the venlafaxine capsules (VEN) were purchased from Wyeth Pharmaceuticals Inc. (Suzhou, China). Purity of the compounds used in the present study were all checked by HPLC and found to be higher than 99%. For the *in vitro* study, LPM580153 were dissolved in dimethyl sulfoxide (DMSO) and for the *in vivo* study, LPM580153 and VEN capsules were suspended with 0.5% sodium carboxymethylcellulose (SCMC) at the proposed dose. Imipramine, protriptyline, GBR12909, 5-HT, NE and DA were all purchased from Sigma (St. Louis, MO, USA). Corticosterone was obtained from EMD Chemicals Inc. (San Diego, CA, USA). Anti-BDNF, anti-ERK1/2 and anti- phosphor-ERK1/2 (Thr202) were bought from Santa Cruz Biotechnology (Santa Cruz, CA, USA). Anti-CREB, anti-phospho-CREB (Ser133), anti-Akt, anti-phospho-Akt (Ser473), anti-mTOR and anti-phospho-mTOR (Ser2448) were obtained from Cell Signaling Technology (Beverly, MA, USA). Anti-PSD95 was purchased from Abcam (Cambridge, MA, USA).

### Animals

Male Swiss mice (18–22 g) and Sprague-Dawley (SD) rats (180–250 g) were obtained from Beijing Weitong Lihua Experimental Animal Centre (Beijing, China). All animals were housed in a light- and temperature-controlled room (12-hour light/dark cycle, lights on at 06:00 h and off at 18:00 h, 21–22 °C, humidity 60–65%) and maintained on a standard diet with continuous access to water. All experiments were performed in accordance with relevant guidelines and regulations approved by the Experimental Animal Research Committee of Yantai University.

### Tail suspension test

The TST protocol was based on a method described previously^23^. Thirty-two Swiss mice were randomly assigned into four groups (n = 8/group): vehicle, VEN (10 mg/kg), and LPM580153 (5 and 10 mg/kg). The animals were fasted overnight with water ad libitum. LPM580153, VEN or vehicle were administrated 60 min before the test. Then, the mice were individually suspended by a hook attached approximately 1 cm from the tip of the tail. The duration of immobility was recorded during the final 4 min of the 6-min test using a polygraph recorder and scored by a blind observer.

### Open field test

Mice were divided and administered the compound as in the TST test. The test was conducted as described previously^12^. Briefly, each animal was placed in one corner of the custom-fabricated activity box (35 × 30 × 22 cm). The distance moved by each mouse was considered an indicator of locomotor activity, and recorded by a polygraph recorder during the final 6 min of the 10-min test.

### Chronic unpredictable mild stress procedure

The rat CUMS model was performed according to previously published methods with minor modifications^23^. Briefly, animals were divided into two groups based on body weight. One group was randomly exposed to various stressors for 3 weeks. The stressors applied included: deprivation of food or water (17 h) or of both water and food deprivation (22 h), overnight illumination, forced cold swimming (40 °C, 5 min), soiled cage (18 h), tail pinch (1 min), white noise (9 h) or 45°cage lit (18 h). No stressor was repeated within 2 days for each rat. The other group was housed in a separate room, with free access to food and water, and not disturbed.

### Sucrose intake test

After 3 weeks of CUMS, sucrose intake test was conducted according to methods described in a previous study with minor modifications^24^. Rats were exposed to 1% sucrose solution for 1 h per day for 3 consecutive days. The test was carried out after 22h of food and water deprivation. Based on the sucrose intake, the CUMS group was divided into five groups and were administered vehicle, VEN (10 mg/kg) or LPM580153 (5, 10 and 20 mg/kg) for another 2 weeks of CUMS. The sucrose intake was performed again after 2 weeks of drug administration.

### Synaptosomal uptake assay

The effects of LPM580153 on the uptake of 5-HT, NE or DA were studied according to published methods^25^. Synaptosomes were made from the frontal cortex, hypothalamus or striatum of the rat brains, and incubated for 15 min at 37 °C with 0.1 μCi ^[3H]^ 5-HT, 0.1 μCi ^[3H]^ NE or 0.1 μCi ^[3H]^ DA in the absence or presence of the test compound or reference compound. Basic control activity was measured in the presence of 10 mM imipramine, 10 mM protriptyline or 1 mM GBR12909, respectively. The reactions were terminated rapidly under vacuum using glass fiber filters (GF/B, Packard) and rinsed twice with ice-cold 50 mM Tris-HCl using a 96-sample cell harvester (Unifilter, Packard). After the filters were dried, sample radioactivity was detected in a scintillation cocktail (Microscint, Packard) using a scintillation counter (Topcount, Packard), and the IC_50_ was calculated.

### SH-SY5Y cell cultures

The neuroblastoma cell line SH-SY5Y (Shanghai Institute of Cell Biology, Chinese Academy of Sciences, Shanghai, China) was maintained in MEM-F12 supplemented with 10% heat-inactivated FBS at 37 °C in an atmosphere of 5% CO_2_ and 95% air. The medium was replaced every 2–3 days.

### Cell viability assay

Cell viability was measured by the 3-(4,5-dimethyl-2-thiazolyl)-2,5-diphenyl-2-H-tetrazolium bromide (MTT) assay. Cells were placed into a 96-well culture plate for 24 h, then exposed to various doses of corticosterone (100, 200, 300, 400 or 500 μM) for another 24 h. To investigate the effect of LPM580153 on the viability of SH-SY5Y cells, cells were treated with LPM580153 (2.5, 5 or 10 μM) for 24 h. To evaluate the protective effect of LPM580153 against corticosterone-induced cell injury, the cells were co-incubated with corticosterone (300 μM) and LPM580153 (2.5, 5 or 10 μM) for 24 h. At the end of treatment, 20 μL MTT (5 mg/ml) was added to each well and incubated for 4 h. The medium was then removed and replaced with 150 μL/well of DMSO to dissolve formazan crystals. Absorbance was measured at 570 nm using a Molecular Devices SpectraMax M5 (Molecular Devices, Foster City, USA). Cell viability was expressed as percentage of the non-treated control. All tests were performed independently three times.

### Western blot analyses

SH-SY5Y cells were seeded into a six-well culture plate for 24 h, then co-incubated with corticosterone (300 μM) and LPM580153 (2.5 or 5 μM) for 24 h. After incubation, cells were rinsed with ice-cold PBS and lysed in RIPA buffer. A tablet of protease inhibitor cocktail (Complete Mini; Roche, San Francisco, CA, USA) was added for each 10 ml of buffer. The cell lysates were centrifuged at 12,000 g for 15 min at 4 °C and the supernatants were collected. Supernatants containing 40 μg of protein were subjected to sodium dodecyl sulfate polyacrylamide gel electrophoresis and transferred to polyvinylidene fluoride membranes. BDNF and the phosphorylated CREB, ERK1/2, AKT and mTOR, were probed with their corresponding specific antibodies and detected using an enhanced chemiluminescence kit (Beyotime Biotechnology, Jiangsu, China), according to the manufacturer’s instructions. Western blots were analysed using Image J software.

### Statistical analysis

Data were presented as mean ± standard deviation (mean ± SD) and were analyzed by SPSS statistical software (version 17.0). Except for data from CUMS test and uptake assays, all other data were analyzed by one-way analysis of variance (ANOVA) followed by Tukey-Kramer test. For data from sucrose intake and body weight in the CUMS test were statistically analyzed by one-way analysis of variance (ANOVA) with repeated measures on the time factor followed by Tukey-Kramer test. For the uptake assay, data were analyzed by LIGAND (Munson and Rodbard) to provide the IC50 values. Significance levels were set at *p* < 0.05.

## Additional Information

**How to cite this article**: Zhang, F. *et al*. Antidepressant-like Effects of LPM580153, A Novel Potent Triple Reuptake Inhibitor. *Sci. Rep.* **6**, 24233; doi: 10.1038/srep24233 (2016).

## Figures and Tables

**Figure 1:**
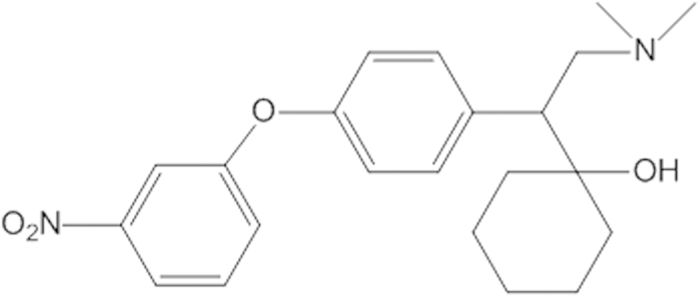
The chemical structure of LPM580153.
